# Effectiveness of diaphragmatic ultrasound as a predictor of successful weaning from mechanical ventilation

**DOI:** 10.1007/s10877-025-01317-8

**Published:** 2025-07-14

**Authors:** Hanady Mohammed Elfeky, Janna Omaran, Noha S. Shaban, Ahmed Elmohamady, Nagwa Doha, Noha Afify

**Affiliations:** 1 Critical Care, Faculty Of Medicine, Menoufia University, Shebeen El-Kom, Egypt; 2 Menoufia University, Faculty of Medicine, Shebeen El-Kom, Egypt

**Keywords:** Diaphragmatic ultrasound, Weaning, Rapid shallow breathing index, Spontaneous breathing trial, Diaphragmatic excursion, Diaphragmatic thickening fraction

## Abstract

**Purpose:** Weaning from mechanical ventilation (MV) is the transition from ventilator dependence to independent breathing. Optimal timing reduces complications. Traditional predictors like the Rapid Shallow Breathing Index (RSBI), diaphragmatic excursion (DE), and diaphragm thickening fraction (DTF) have limitations. This study evaluates the clinical utility of diaphragmatic excursion RSBI (DE-RSBI) and diaphragm thickening fraction RSBI (DTF-RSBI) alongside conventional RSBI in predicting weaning success. **Methods:** An observational study was conducted in the ICU of Menoufia University Hospitals on 50 adult patients mechanically ventilated for over 48 hours who underwent spontaneous breathing trials (SBT). Diaphragmatic ultrasound measured DE and DTF, from which DE-RSBI and DTF-RSBI were calculated and compared with RSBI. Weaning success was defined as maintaining spontaneous breathing for more than 48 hours post-extubation. **Results:** DE-RSBI (≥1.685) showed the highest predictive accuracy (AUC=0.851, sensitivity=80%, specificity=85%). Both DE-RSBI and DTF-RSBI correlated with ICU mortality (p<0.001) and MV duration (p≤0.001). Weaning failure odds were significantly higher for DE-RSBI >1.56 (OR=12, p=0.004) and DTF-RSBI >62.33 (OR=8.04, p=0.008) compared to RSBI alone (OR=4.84, p=0.04). **Conclusions:** DE-RSBI and DTF-RSBI are reliable predictors of MV weaning, outperforming RSBI alone. Their use can enhance weaning decisions, reducing failure rates and improving patient outcomes.

## Introduction

 Weaning from mechanical ventilation (MV) is a critical phase in intensive care management. Delays in weaning are associated with serious complications such as ventilator-associated pneumonia and ventilator-induced diaphragmatic atrophy [1]. Although standardized weaning criteria exist, approximately 20% of extubated patients experience failure, underscoring the need for more accurate predictors to reduce morbidity and mortality [2].

The spontaneous breathing trial (SBT), typically performed using pressure support ventilation (PSV), remains the standard method to initiate weaning [3]. Weaning failure is defined as the inability to tolerate the SBT or the requirement for reintubation within 48 h after extubation [4]. Diaphragmatic dysfunction, a major contributor to weaning failure, may result from prolonged MV, neuromuscular blockade, or sepsis [5–7]. Clinically, diaphragmatic dysfunction may manifest as respiratory distress, hypercapnia, or hypoxemia, and up to 25% of patients may require reintubation or noninvasive ventilation (NIV) [8].

Diaphragmatic ultrasound has emerged as a reliable, non-invasive method for real-time assessment of diaphragm function. It allows measurement of diaphragmatic excursion (DE), thickness, contractility, and thickening fraction (DTF), offering valuable insights into weaning readiness [9]. Predictive indices such as the rapid shallow breathing index (RSBI), vital capacity (VC), DE, DTF, and maximal inspiratory pressure (PIMAX) have all been studied for their association with weaning outcomes [10]. A DE < 10 mm has been linked to weaning failure [11], whereas a DTF > 20% is associated with successful weaning [12].

RSBI is traditionally considered one of the most reliable weaning predictors. However, its accuracy was limited by various physiological and mechanical factors [13]. During PSV, accessory muscle use may compensate for diaphragmatic weakness, potentially concealing dysfunction [14].

To overcome these limitations, modified indices such as diaphragmatic excursion-RSBI (DE-RSBI) and diaphragmatic thickening fraction-RSBI (DTF-RSBI) have been proposed [15]. Early evidence suggests that integrating ultrasound-derived indices with traditional weaning criteria may improve predictive accuracy [16].

This study aims to evaluate the effectiveness of two ultrasound-based indices—DE-RSBI and DTF-RSBI—in predicting successful weaning from mechanical ventilation (MV). These indices will be compared with the traditional RSBI during SBTs to support more accurate clinical decision-making.

## Methods

### Study design

This prospective observational study was conducted in the Intensive Care Unit (ICU) of Menoufia University Hospital, with ethical approval obtained from the Institutional Review Board (IRB) of Menoufia University, Shibin El Kom, Egypt (Approval No. 09/2023 ANET 30) in September 2023. The study included 50 mechanically ventilated patients aged between 15 and 65 years. Participants were categorized into two groups based on weaning outcomes: the success group (*n* = 40) and the failure group (*n* = 10). Written informed consent was obtained from the legal surrogates of all participants before enrollment.

### Sample size

Based on prior research reporting an area under the receiver operating characteristic (ROC) curve (AUC) of 0.97 (*p* < 0.0001) for diaphragmatic RSBI indices, the required sample size was calculated using Statistical and Sample Size Pro software, version 6. A total of 50 subjects was determined to be adequate to achieve a study power of 80% with a confidence level of 95% [17].

### Study population

The study population consisted of patients who had undergone intubation and mechanical ventilation for over 48 h and met the ICU’s standard weaning criteria for initiating the SBT. Eligibility requires resolution or significant improvement of the initial condition necessitating mechanical ventilation, along with adequate oxygenation.

Adequate oxygenation was defined as arterial oxygen saturation (SaO₂) above 90% while receiving an inspired oxygen fraction (FiO₂) of 0.5 or less, or a PaO₂/FiO₂ ratio greater than 200 mmHg, both maintained with a positive end-expiratory pressure (PEEP) ≤ 8 cmH₂O. Additional inclusion criteria included a respiratory rate of ≤ 30 breaths per minute, a preserved cough reflex, and hemodynamic stability with minimal or no vasopressor support.

Exclusion criteria included pregnancy, thoracostomy or pneumothorax, flail chest or rib fractures, neuromuscular disorders, diaphragmatic paralysis, and cerebrovascular stroke with bulbar symptoms. Only patients who met all inclusion criteria and none of the exclusion criteria were enrolled in the study.

### Outcome definitions

Successful weaning was defined as the ability to sustain spontaneous breathing for more than 48 h after extubation without requiring NIV or reintubation. Conversely, weaning failure was defined as the need for reintubation or NIV within 48 h of extubation [4].

Clinical data—including age, Acute Physiology and Chronic Health Evaluation II (APACHE II) score, Sequential Organ Failure Assessment (SOFA) score on the day of ICU admission, total ICU stay, and duration of mechanical ventilation—were recorded. Decisions regarding SBT initiation, extubation, or reinstitution of ventilation were made by the attending physician based on clinical judgment.

### Study methods

Eligible patients underwent a SBT using PSV, with a pressure support level of 5 cmH₂O above a PEEP of 5 cmH₂O, resulting in a total inspiratory pressure of 10 cmH₂O. The trial lasted between 30 and 60 min.

Both the conventional RSBI and DE were measured at two time points: at the beginning and the end of the SBT. Other parameters, including DTF, DE-RSBI, and DTF-RSBI, were measured at the end of the SBT.

Ultrasound assessments followed a standardized protocol and were conducted by an ICU physician experienced in ultrasonography. The physician conducting the ultrasound assessments was not involved in patient care and was blinded to the clinical outcomes. Similarly, attending physicians, who were blinded to the ultrasound results, made extubation decisions based solely on clinical judgment.

Patients who maintained spontaneous breathing beyond 48 h post-extubation were assigned to the success group, while those requiring reintubation or NIV during this period comprised the failure group.

The conventional RSBI was calculated by dividing the respiratory rate (RR) by VT (RSBI = RR/VT).

### Diaphragm ultrasound

A 5 MHz curvilinear probe in two-dimensional (2D) mode was used to ensure optimal imaging quality. DE was assessed by placing the probe over the lower intercostal spaces in the right mid-axillary line, using the liver as an acoustic window to visualize the right hemidiaphragm. DE was measured as the vertical amplitude of diaphragmatic motion from baseline to peak inspiration.

The right hemidiaphragm was also visualized at the zone of apposition with the rib cage, located between the 8th and 10th intercostal spaces along the mid-axillary line. Diaphragm thickness (DT) was measured at end-inspiration and end-expiration. Diaphragm thickening difference (DTD) was calculated by subtracting DT at end-expiration from DT at end-inspiration, and the DTF was derived using the formula:

DTF = (DTD / DT at end-expiration) × 100%.

Ultrasound-derived diaphragmatic RSBI parameters were calculated by replacing VT in the traditional RSBI formula with ultrasound-based measurements. Specifically, DE-RSBI was calculated as RR divided by DE, and DTF-RSBI as RR divided by DTF.

### Outcomes

Data were collected for all patients undergoing the SBT, including sex, age, weight, and comorbidities such as hypertension, diabetes mellitus, heart disease, and renal or hepatic disorders. Documented clinical characteristics included mean arterial pressure, heart rate, oxygen saturation, respiratory rate, and tidal volume.

During the SBT, the following parameters were assessed: RSBI (initial and end), DE (initial and end), and diaphragmatic thickness at end-inspiration and end-expiration, which was used to calculate DTF.

Ultrasound-derived RSBI parameters were also derived, including DE-RSB and DTF-RSBI at the end of SBT.

### Statistical analysis

Data were collected, tabulated, and statistically analyzed using SPSS software (Version 22) on an IBM-compatible personal computer. Quantitative data were presented as medians and interquartile ranges, along with means ± standard deviation (SD), while percentages and frequencies were used for categorical data. Student’s t-tests were used for comparisons of symmetrically distributed parameters, while skewed continuous variables were analyzed using the Mann-Whitney-Wilcoxon rank-sum test for independent samples and the Wilcoxon signed-rank test for paired samples. Categorical data were assessed using the Chi-square test.

Correlation analysis was performed using Pearson and Spearman’s correlation coefficients. Logistic regression analysis was used to identify factors associated with weaning failure. Continuous variables were categorized using either the mean or median as a cutoff, depending on their distribution. Variables with a univariate p-value ≤ 0.1 were entered into a multivariate logistic regression model. A p-value < 0.05 was considered statistically significant.

For receiver operating characteristic (ROC) curve analysis, the area under the curve (AUC) was computed and presented with a 95% confidence interval (CI). Since ROC analysis is inherently non-parametric, it was unaffected by distribution skewness and invariant to log transformation. The positive predictive value (PPV), negative predictive value (NPV), sensitivity, specificity, optimal cutoff point, and accuracy were calculated for each ROC curve.

## Results

**Fig. 1:**
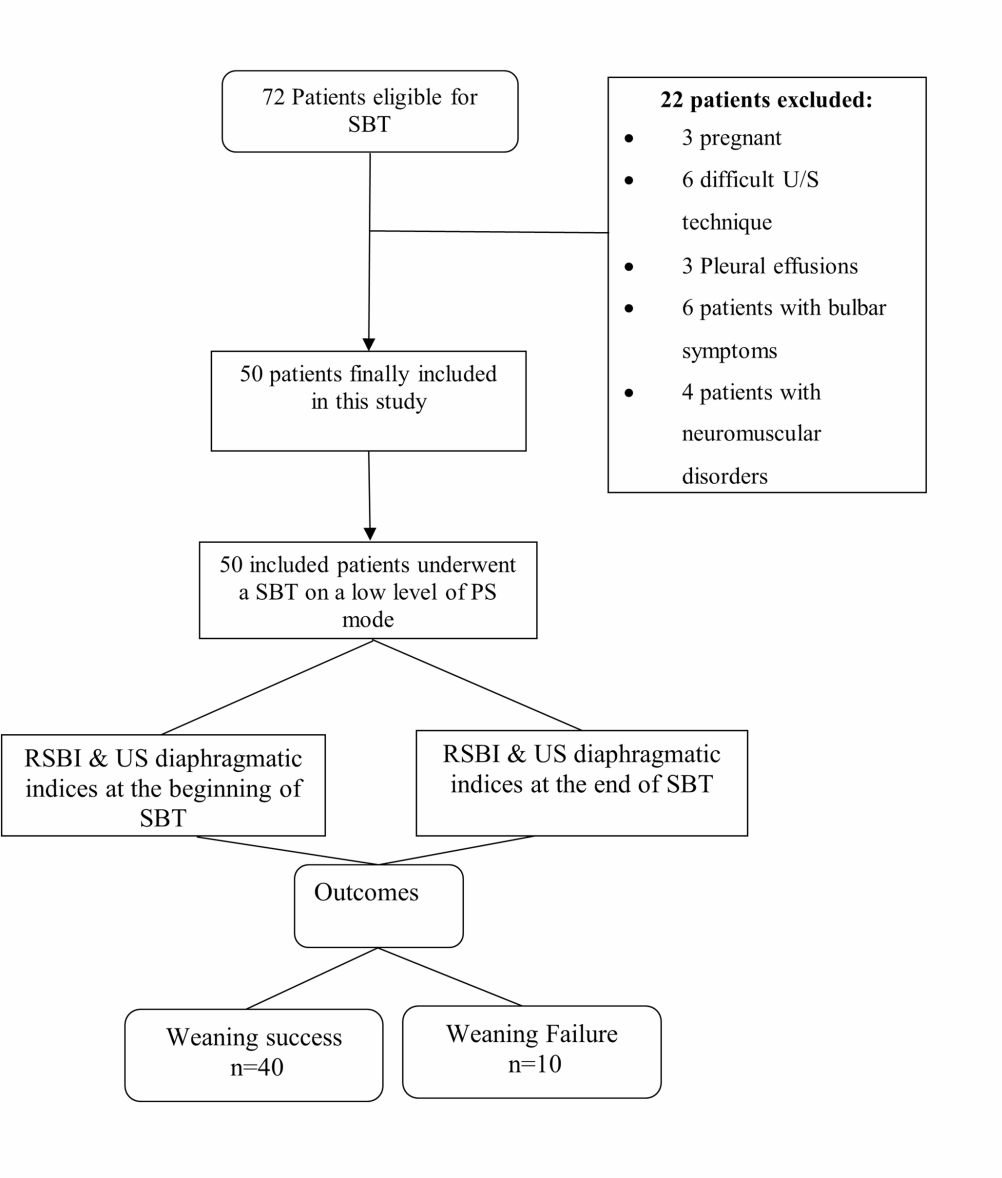
Flowchart of the study

This prospective study included 50 adult patients admitted to the intensive care units of Menoufia University Hospitals who underwent a SBT and were assessed for weaning from mechanical ventilation. Patients were categorized into two groups: successful weaning (*n* = 40) and failed weaning (*n* = 10). A total of 22 patients were excluded for various reasons, including pregnancy (*n* = 3), poor ultrasound window (*n* = 6), presence of pleural effusion (*n* = 3), bulbar symptoms (*n* = 6), and neuromuscular disorders (*n* = 4). Figure 1 presents the study flowchart.

Table 1 summarizes the demographic characteristics, comorbidities, primary indications for mechanical ventilation, and clinical outcomes. The mean age was 56.58 ± 10.81 years (range: 19–65 years), with a statistically significant age difference between the weaning success and failure groups (*p* = 0.034). No significant differences were observed between groups regarding sex distribution (*p* = 0.480) or body mass index (*p* = 0.125). The most common comorbidities were hypertension (54%), diabetes mellitus (50%), and ischemic heart disease (28%), with no significant intergroup differences.

**Table 1:** Demographic characteristics, comorbidities, primary causes of ventilation, and clinical outcomes in both study groups, weaning success (*n* = 40) and failure (*n* = 10)

Variables		All patients (*N* = 50)	Weaning Success group (*n* = 40)	Weaning Failure group (*n* = 10)	Test of significance	*P* value
**Age (years)**	**Mean ± SD** **Min-Max**	56.58 ± 10.81 (19–65)	55.57 ± 11.72 (19–65)	60.60 ± 4.24 (52–65)	t = 2.19	0.034*
**Sex**	**Male**,** N (%)** **Female**,** N (%)**	27(54%) 23(46%)	23(57.5%) 17(42.5%)	4(40%) 6(60%)	χ2 = 0.98	0.480
**BMI**	**Mean ± SD** **Min-Max**	24.60 ± 2.98 (19–31)	24.27 ± 2.50 (19–28)	25.90 ± 4.38 (19–31)	t = 1.56	0.125
**Comorbidities N (%)**						
**HTN**		27(54%)	22(55%)	5(50%)	χ2 = 0.05	1
**DM**		25(50%)	19(47.5%)	6(60%)	FE = 0.50	0.725
**IHD**		14(28%)	12(30%)	2(20%)	FE = 0.39	0.704
**Stroke**		6(12%)	6(15%)	0	FE = 1.71	0.327
**CKD**		7(14%)	4(10%)	3(30%)	FE = 2.65	0.133
**Primary causes of MV (N% %)**						
**Pneumonia**		11(22%)	10(25%)	1(10%)	FE = 10.56	0.566
**Type 2 RF**		3(6%)	1(2.5%)	2(20%)		
**Septic shock**		10(20%)	7(17.5%)	3(30%)		
**Hemorrhagic Shock**		1(2%)	1(2.5)	0		
**Pulmonary edema**		6(12%)	6(15%)	0		
**Asthma Exacerbation**		1(2%)	1(2.5%)	0		
**DCL**		5(10%)	4(10%)	1(10%)		
**AKI**		9(18%)	6(15%)	3(30%)		
**Cerebral Hemorrhage**		1(2%)	1(2.5%)	0		
**Seizures**		1(2%)	1(2.5%)	0		
**Snake bite**		1(2%)	1(2.5%)	0		
**Diabetic- keto acidosis**		1(2%)	1(2.5%)	0		
**Clinical Scores Mean ± SD (Min–Max)**						
**APACHE II score**		21.36 ± 5.69 (11–33)	19.97 ± 5.22 (11–32)	26.90 ± 3.92 (23–33)	t = 4.64	< 0.001*
**SOFA score on admission**		7.74 ± 2.13 (4–14)	7.40 ± 1.98 (4–11)	9.10 ± 2.28 (7–14)	t = 2.16	0.051
**Sofa Score 7th day**		3.04 ± 1.73 (0–8)	2.45 ± 1.23 (0–5)	5.40 ± 1.42 (3–8)	t = 5.98	< 0.001*
**Clinical Outcomes Mean ± SD (Min–Max) or N (%)**						
**Days of MV**		5.16 ± 1.88 (2–10)	4.55 ± 1.41 (2–8)	7.60 ± 1.57 (5–10)	t = 5.57	< 0.001*
**Length of ICU Stay**		10.56 ± 3.17 (5–18)	9.77 ± 2.73 (5–15)	13.7 ± 2.9 (8–16)	t = 3.78	0.002*
**7th -day Mortality (N=%)**		0	0	0	-	-
**28-day Mortality (N=%)**		10 (	2(5%)	8(80%)	χ2 = 28.13	< 0.001*

SOFA, Sequential Organ Failure Assessment. ICU, intensive care unit. MV, mechanical ventilation. APACHE II, Acute Physiology and Chronic Health Evaluation II. AKI, acute kidney injury. DCL, disturbed consciousness level. RF, respiratory failure. HTN, hypertension. DM, diabetes mellitus. CKD, chronic kidney disease. IHD, ischemic heart disease. BMI, body mass index. SD, standard deviation. Min, minimum. Max, maximum. (t) Student t-test, (FE): Fisher exact test, χ², (Chi-square) test. (*) statistically significant. N, number

The primary indications for mechanical ventilation were pneumonia (22%), septic shock (20%), and acute kidney injury (18%). These indications did not significantly differ between the two groups.

**Fig. 2:**
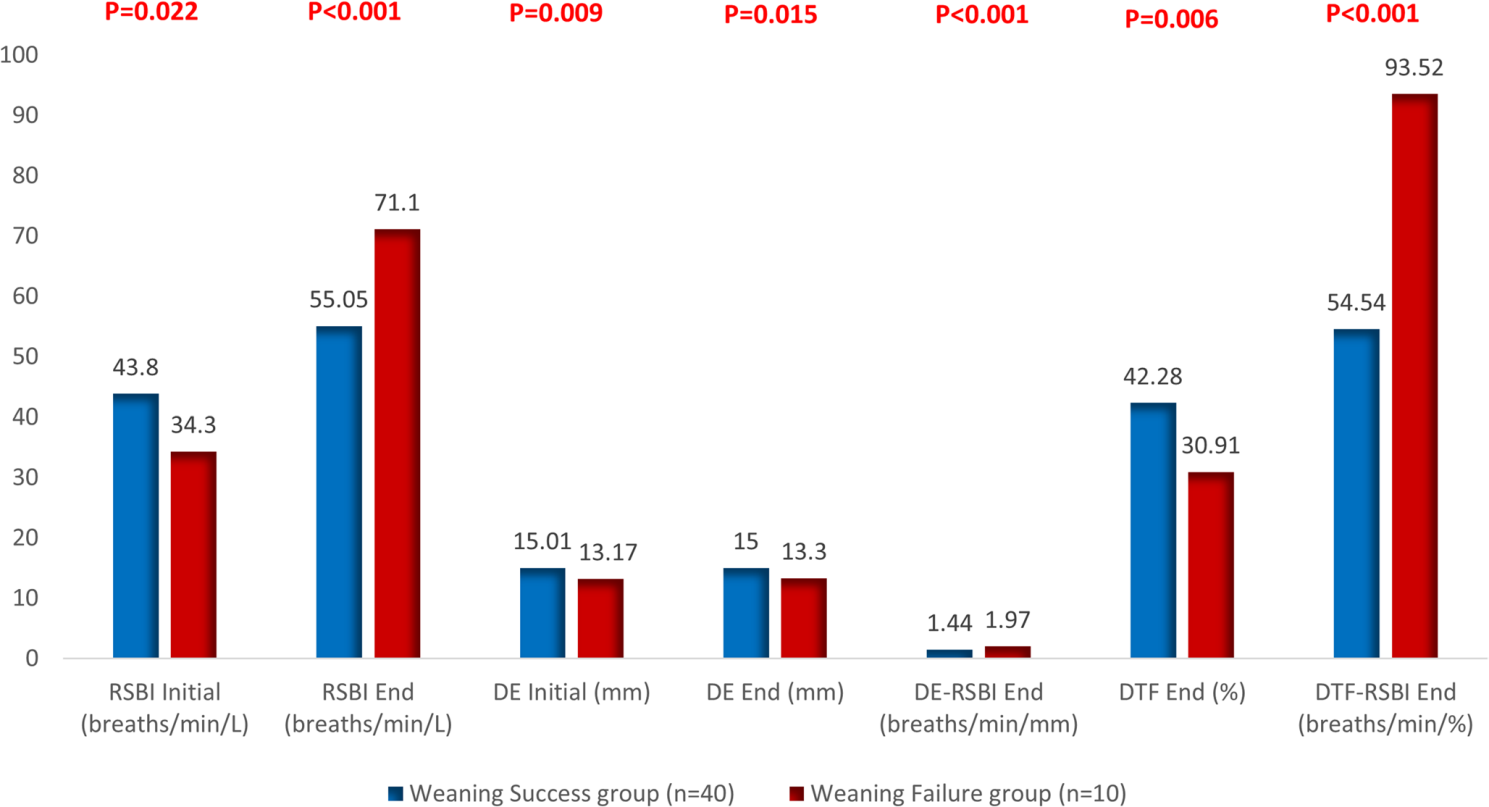
RSBI and ultrasound-derived diaphragmatic parameters in both study groups, weaning success (*n* = 40) and failure (*n* = 10)

Clinical severity scores showed notable intergroup differences. The weaning failure group had significantly higher APACHE II scores (26.90 ± 3.92) compared to the success group (19.97 ± 5.22; *p* < 0.001). Similarly, SOFA scores on day 7 were significantly elevated in the failure group (5.40 ± 1.42) compared to the success group (2.45 ± 1.23; *p* < 0.001).

Regarding clinical outcomes, the weaning failure group had a significantly longer duration of mechanical ventilation (7.60 ± 1.57 vs. 4.55 ± 1.41 days, *p* < 0.001) and ICU stay (13.7 ± 2.9 vs. 9.77 ± 2.73 days, *p* = 0.002). The 28-day mortality rate was significantly higher in the failure group (80%) than in the success group (5%; *p* < 0.001).

Hemodynamic parameters at the start of the SBT did not differ significantly between groups. However, by the end of the SBT, significant differences emerged in mean arterial pressure (*p* = 0.001), heart rate (*p* = 0.001), respiratory rate (*p* < 0.001), SpO₂ (*p* = 0.001), and FiO₂ (*p* < 0.001), as detailed in Table 2.

**Table 2:** Comparisons of hemodynamics characteristics at the beginning and the end of SBT in both study groups, weaning success (*n* = 40) and failure (*n* = 10)

Variables	Weaning Success group (*n* = 40)	Weaning Failure group (*n* = 10)	Test of significance	*P* value
**Hemodynamics at the beginning of SBT**				
**MAP (mmHg)** **Mean ± SD (Min–Max)**	74.87 ± 6.83 (65–90)	73.70 ± 5.88 (65–85)	t = 0.54	0.593
**HR (beats/minute)** **Mean ± SD (Min–Max)**	80.05 ± 9.62 (65–97)	80.00 ± 9.71 (64–92)	t = 0.02	0.989
**RR (breaths/minute)** **Mean ± SD (Min–Max)**	21.93 ± 2.26 (18–27)	21.4 ± 2.54 (18–26)	t = 0.59	0.562
**SPO2%** **Mean ± SD (Min–Max)**	96.17 ± 1.33 (94–99)	95.8 ± 1.31 (94–98)	t = 0.80	0.435
**FIO2%** **Mean ± SD (Min–Max)**	36.5 ± 2.32 (35–40)	37.5 ± 3.53 (35–45)	t = 1.09	0.281
**Vasopressors (N% %)**	7 (17.5%)	3 (30%)	FE = 0.78	0.397
**Hemodynamics after the End of SBT**				
**MAP (mmHg)** **Mean ± SD (Min–Max)**	73.12 ± 5.02 (65–85)	64.5 ± 5.50 (55–70)	t = 4.51	0.001*
**HR (beat/minute)** **Mean ± SD (Min–Max)**	83.85 ± 7.70 (69–97)	103.4 ± 13.25 (89–135)	t = 4.47	0.001*
**RR (rate/minute)** **Mean ± SD (Min–Max)**	21.67 ± 1.76 (18–25)	25.80 ± 3.85 (19–30)	t = 5.07	< 0.001*
**SPO2%** **Mean ± SD (Min–Max)**	95.75 ± 1.17 (92–98)	93.2 ± 2.48 (88–96)	t = 3.47	0.001*
**FIO2%** **Mean ± SD (Min–Max)**	33.95 ± 7.46 (21–45)	51.5 ± 5.29 (45–60)	t = 8.56	< 0.001*
**Vasopressors (N% %)**	7(17.5%)	4(40%)	FE = 3.36	0.197

SBT, Spontenous breathing trial. MAB, mean arterial pressure. mmHg, millimeters of mercury. HR, heart rate. RR, respiratory rate. SPO2, peripheral capillary oxygen saturation. FIO2, Fraction of Inspired Oxygen. N, number. SD, standard deviation. Min, minimum. Max, maximum. (t) Student t test, (FE): Fisher exact test, (*): statistically significant

Ultrasound-based weaning parameters are shown in Table 3; Fig. 2. At the beginning of the SBT, the RSBI was significantly lower in the failure group (34.30 ± 10.48 breaths/min/L) compared to the success group (43.80 ± 9.31 breaths/min/L; *p* = 0.022). By the end of the trial, RSBI values increased more in the failure group (71.10 ± 19.31 vs. 55.05 ± 7.32 breaths/min/L; *p* < 0.001). Both DE (initial and end) and DTF were significantly reduced in the failure group (*p* < 0.05). Additionally, the DE-RSBI and DTF-RSBI indices were significantly elevated in the failure group (*p* < 0.001).

**Table 3:** Comparison of RSBI and diaphragm ultrasound-derived parameters in both study groups, weaning success (*n* = 40) and failure (*n* = 10)

Variables	All patients (*N* = 50)	Weaning Success group (*n* = 40)	Weaning Failure group (*n* = 10)	Test of significance	*P* value
**RSBI initial (breaths/min/L)**	41.90 ± 10.19 19–60	43.80 ± 9.31 29–60	34.30 ± 10.48 19–52	t = 2.62	0.022*
**RSBI End** **(breaths/min/L)**	85.26 ± 12.38 35—98	55.05 ± 7.32 35–70	71.10 ± 19.31 41–98	t = 4.26	< 0.001*
**DE initial (mm)**	14.64 ± 1.63 10.8—17.8	15.01 ± 1.39 11.1–17.8	13.17 ± 1.75 10.8–16.1	t = 3.08	0.009*
**DE End (mm)**	14.72 ± 1.75 10.8—18.2	15.08 ± 1.56 12.4–18.2	13.30 ± 1.84 10.8–16.2	t = 2.81	0.015*
**DE-RSBI End** **(breaths/min/mm)**	1.56 ± 0.34 1.17—2.77	1.44 ± 0.21 1.17–1.93	1.97 ± 0.45 1.32–2.77	t = 13.97	< 0.001*
**DTF End (%)**	40.01 ± 10.89 19—60	42.28 ± 9.97 20.8–60	30.91 ± 10.04 19–45	t = 3.21	0.006*
**DTF-RSBI End (breath /min/%)**	62.33 ± 26.39 31.6—150	54.54 ± 15.78\ 31.6–105	93.52 ± 36.90 42–150	t = 5.48	< 0.001*

RSBI, rapid shallow breathing index. DE, diaphragmatic excursion. DE-RSBI, diaphragmatic excursion rapid shallow breathing index. DTF, diaphragmatic thickening fraction. DTF-RSBI, diaphragmatic thickening fracture rapid shallow breathing index. (t) Student t-test, (*): statistically significant

**Fig. 3:**
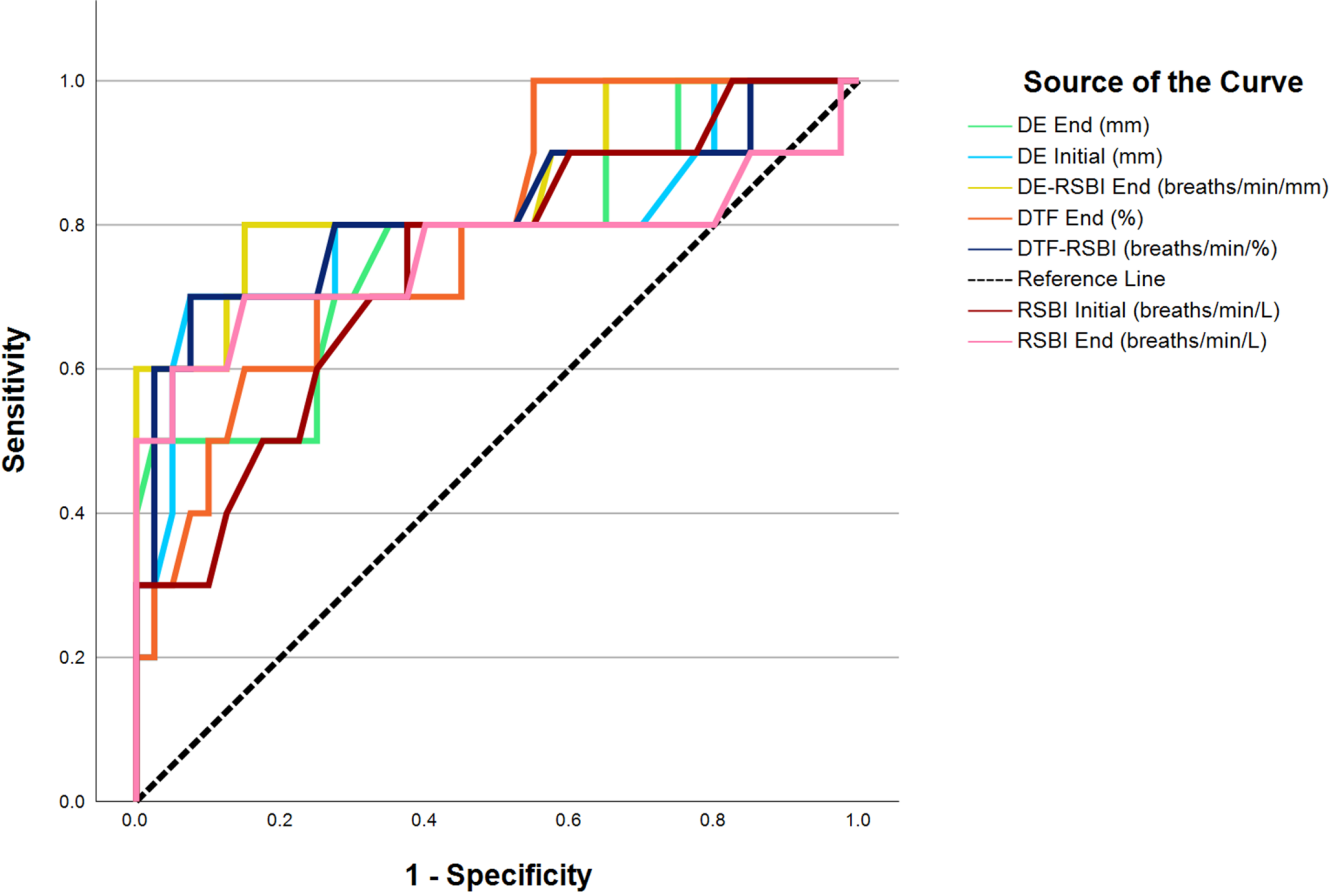
The ROC curve for RSBI and ultrasound-derived diaphragmatic parameters

Univariate logistic regression analysis identified RSBI > 58.26, DE ≤ 14.72, DE-RSBI > 1.56, and DTF-RSBI > 62.33 as significant predictors of weaning failure (*p* < 0.05). However, in multivariate logistic regression analysis, none of these parameters remained statistically significant independent predictors, as shown in Table 4.

**Table 4:** Univariate and multivariate logistic regression for weaning failure (*n* = 10)

Variables	Univariate logistic regression			Multivariate logistic regression		
	Odds ratio	*P* value	CI = 95%	Odds ratio	*P* value	CI = 95%
**RSBI initial (> 41.9)**	4.89	0.063	0.92–25.97	-	-	-
**RSBI End (> 85.26)**	4.84	0.040*	1.07–21.84	1.65	0.655	0.18–15.28
**DE initial (≤ 14.64)**	6.67	0.027*	1.24–35.6	4.91	0.168	0.51–47.12
**DE End (≤ 14.72)**	6.67	0.027*	1.24–35.6	1.47	0.804	0.07–30.84
**DE-RSBI (> 1.56)**	12	0.004*	2.18–66.13	4.37	0.354	0.19–98.82
**DTF End (< 40.01)**	3.16	0.131	0.71-14.0	-	-	-
**DTF-RSBI (> 62.33)**	8.04	0.008*	1.71–37.59	5.22	0.071	0.87–31.44

RSBI, rapid shallow breathing index. DE, diaphragmatic excursion. DE-RSBI, diaphragmatic excursion rapid shallow breathing index. DTF, diaphragmatic thickening fraction. DTF-RSBI, diaphragmatic thickening fracture rapid shallow breathing index. CI, confidence interval

The predictive performance of RSBI, DE, and DTF for weaning failure is summarized in Table 5; Fig. 3. The DE-RSBI demonstrated the highest predictive accuracy (AUC = 0.851, *p* < 0.001), followed by DTF-RSBI (AUC = 0.796, *p* = 0.002) and RSBI at the end of the trial (AUC = 0.763, *p* = 0.020). These findings support the utility of diaphragmatic ultrasound-derived indices, particularly DE-RSBI and DTF-RSBI, in predicting weaning failure, though further validation in larger cohorts is warranted.

**Table 5:** Predictive accuracy of RSBI and ultrasound-derived diaphragmatic parameters for weaning failure

Variable	AUC	Cut-Off Point	*P* value	Sensitivity (%)	Specificity (%)	Accuracy (%)	PPV (%)	NPV (%)
**RSBI initial** (breaths/min/L)	0.746 (0.572–0.921)	≤ 40.5	0.006*	80%	62.5%	66%	34.8%	92.6%
**RSBI End** (breaths/min/L)	0.763 (0.541–0.984)	≥ 63	0.020*	70%	85%	82%	53.8%	91.9%
**DE initial (mm)**	0.796 (0.607–0.985)	≤ 13.25	0.002*	70%	92.5%	88%	70%	92.5%
**DE End (mm)**	0.775 (0.598–0.952)	≤ 14.55	0.002*	80%	65%	68%	36.4%	92.9%
**DE-RSBI End** (breaths/min/mm)	0.851 (0.698–1.005)	≥ 1.685	< 0.001*	80%	85%	84%	57.1%	94.4%
**DTF End (%)**	0.789 (0.638–0.939)	≤ 35.5	< 0.001*	70%	75%	74%	41.2%	90.9%
**DTF-RSBI** (breaths/min/%)	0.819 (0.64–0.998)	≥ 81.75	< 0.001*	70%	92.5%	88%	70%	92.5%

RSBI, rapid shallow breathing index. DE, diaphragmatic excursion. DE-RSBI, diaphragmatic excursion rapid shallow breathing index. DTF, diaphragmatic thickening fraction. DTF-RSBI, diaphragmatic thickening fracture rapid shallow breathing index. AUC, area under the curve. PPV, positive predictive value. NPV, negative predictive value

Correlational analysis revealed that the days of invasive mechanical ventilation were significantly and positively associated with RSBI at the end of SBT (r = + 0.44, *p* = 0.001), DE-RSBI (r = + 0.46, *p* = 0.001), and DTF-RSBI (r = + 0.49, *p* < 0.001). A significant negative correlation was found with DTF at the end of SBT (*r* = − 0.29, *p* = 0.043). ICU length of stay also correlated positively with DTF-RSBI (r = + 0.28, *p* = 0.049). For ICU mortality, there were positive correlations with RSBI end (r = + 0.55, *p* < 0.001), DE-RSBI (r = + 0.60, *p* < 0.001), and DTF-RSBI (r = + 0.62, *p* < 0.001), and negative correlations with DE initial (*r* = − 0.52, *p* < 0.001), DE end (*r* = − 0.36, *p* = 0.009), and DTF end (*r* = − 0.47, *p* = 0.001), as shown in Table 6.

**Table 6:** Correlation between RSBI and Ultrasound-derived diaphragmatic indices with days of invasive mechanical ventilation, length of stay in the ICU, and ICU mortality

Variable	Days of invasive mechanical ventilation		length of stay in the ICU		ICU mortality	
	*r*	*P* value	*r*	*P* value	*r*	*P* value
**RSBI initial** **breaths/min/L**	-0.23	0.101	-0.12	0.412	-0.13	0.373
**RSBI End** **breaths/min/L**	+ 0.44	0.001*	+ 0.19	0.196	+ 0.55	< 0.001*
**DE initial (mm)**	-0.26	0.071	-0.19	0.17	-0.52	< 0.001*
**DE End (mm)**	-0.16	0.284	-0.09	0.535	-0.36	0.009*
**DE-RSBI** **breaths/min/mm**	+ 0.46	0.001*	+ 0.25	0.083	+ 0.60	< 0.001*
**DTF End (%)**	-0.29	0.043*	-0.21	0.148	-0.47	0.001*
**DTF-RSBI** **breaths/min/%**	+ 0.49	< 0.001*	+ 0.28	0.049*	+ 0.62	< 0.001*

RSBI, rapid shallow breathing index. DE, diaphragmatic excursion. DE-RSBI, diaphragmatic excursion rapid shallow breathing index. DTF, diaphragmatic thickening fraction. DTF-RSBI, diaphragmatic thickening fracture rapid shallow breathing index. AUC, area under the curve. PPV, positive predictive value; NPV, negative predictive value. ICU, intensive care unit. r, correlation coefficient. (*) statistically significant

SBT, Spontaneous Breathing Trial; PS, Pressure Support; US, Ultrasound; RSBI, Rapid Shallow Breathing Index

RSBI, rapid shallow breathing index. DE, diaphragmatic excursion. DE-RSBI, diaphragmatic excursion rapid shallow breathing index. DTF, diaphragmatic thickening fraction. DTF-RSBI, diaphragmatic thickening fracture rapid shallow breathing index. Statistically significant at *P* < 0.05

ROC, Receiver operating characteristic curve. RSBI, rapid shallow breathing index. DE, diaphragmatic excursion. DE-RSBI, diaphragmatic excursion rapid shallow breathing index. DTF, diaphragmatic thickening fraction. DTF-RSBI, diaphragmatic thickening fracture rapid shallow breathing index

## Discussion

Weaning patients from mechanical ventilation in the ICU is a complex process influenced by both demographic and clinical factors. Studies indicate that the weaning failure rate among ICU patients is around 20% [18], a finding consistent with our results. Pneumonia (22%) was the most common cause of mechanical ventilation, followed by septic shock (20%) and acute kidney injury (18%). The most common comorbidities were hypertension (54%), diabetes mellitus (50%), and ischemic heart disease (28%).

Patients in the failed weaning group had significantly higher severity scores (APACHE II: 26.90 ± 3.92 vs. 19.97 ± 5.22, *p* < 0.001; SOFA: 5.40 ± 1.42 vs. 2.45 ± 1.23, *p* < 0.001), longer ICU stays (13.7 ± 2.9 vs. 9.77 ± 2.73 days, *p* = 0.002), and a higher 28-day mortality rate (80% vs. 5%, *p* < 0.001).

Other demographic variables did not differ significantly between the groups. These findings are consistent with previous studies that link weaning failure to baseline conditions such as cardiac dysfunction, muscle weakness, pulmonary insufficiency, and electrolyte disturbances [19]. However, our study found no direct association between these factors and weaning failure. While hemodynamic parameters at the start of the SBT showed no significant differences, all parameters became statistically significant by the trial’s end (*p* ≤ 0.001), highlighting their predictive value for weaning success.

The RSBI is widely used to predict weaning success, as it evaluates respiratory efficiency by measuring the ratio of frequency to TV [13]. Previous studies have demonstrated that successful extubation occurs with an RSBI cutoff of 59, showing a sensitivity of 79% and specificity of 64%, which is consistent with our findings [20]. Another study reported a sensitivity of 97% and a specificity of 64% for RSBI [21].

Karthika M et al. identified 105 breaths/min as the traditional cutoff point in clinical trials, with a sensitivity of 97% and specificity of 64% [22]. In contrast to the traditional RSBI, Spadaro et al. showed that substituting diaphragmatic ultrasound parameters for tidal volume when calculating D-RSBI enhances the prediction capacity for weaning [21]. One study demonstrated that using diaphragmatic RSBI results in a sensitivity of 97.3% and a specificity of 93.9%, highlighting its superiority over traditional RSBI [17].

Further studies suggest that weaning success may be influenced by increased inspiratory time, as a reduced inspiratory flow can decrease the work of breathing. Palkar et al. introduced the Excursion-Time (E-T) index as a measure that reflects diaphragmatic performance and workload during inspiration [23, 24]. This highlights a key limitation of the RSBI, which does not account for the underlying pathophysiological causes of weaning failure. RSBI may also be confounded by accessory muscle use, potentially masking diaphragmatic dysfunction.

In our study, the RSBI measured at the start of the SBT demonstrated a sensitivity of 80% and specificity of 62.5% (AUC = 0.746). By the end of the SBT, sensitivity and specificity improved to 70% and 85%, respectively (AUC = 0.763), highlighting RSBI’s potential as a strong predictor of weaning success. However, due to variability in patient populations, outcome definitions, and measurement techniques across studies, RSBI alone cannot be solely relied upon.

Diaphragmatic excursion, commonly assessed via ultrasound, is a key parameter for evaluating diaphragm movement during the respiratory cycle [25]. It plays an important role in predicting weaning outcomes, as it quantifies diaphragmatic motion and assists in diagnosing conditions linked to diaphragmatic dysfunction—such as phrenic nerve palsy, neuromuscular disorders, and chronic obstructive pulmonary disease (COPD)—which are frequently encountered in ICU patients [26, 27].

A meta-analysis by Parada-Greda et al. reported that DE had a sensitivity and specificity of 80%, with an AUC of 0.87 for predicting weaning failure [28]. Similarly, a study by Rui Li et al. demonstrated a sensitivity of 90.2%, specificity of 60%, and an AUC of 0.785 [29].

In our study, DE measured at the start of the SBT showed a sensitivity of 70% and specificity of 92.5% (AUC = 0.796) at a cutoff value of ≤ 13.25 mm. When measured at the end of the SBT, DE demonstrated a sensitivity of 80% and specificity of 65% (AUC = 0.755) at a cutoff of ≤ 14.55 mm. These findings are consistent with previous studies, further supporting DE as a reliable marker of diaphragmatic function and predictor of weaning outcomes.

DTF is an ultrasound-based parameter that quantifies the percentage change in diaphragm thickness between end-expiration and end-inspiration. It is measured at the zone of apposition of the diaphragm to the rib cage using ultrasound [30, 31]. Higher DTF values reflect greater diaphragmatic contractility and are often associated with successful weaning, whereas lower values indicate diaphragmatic dysfunction and an increased risk of weaning failure [32, 33].

In the same meta-analysis by Parada-Greda et al., DTF demonstrated a sensitivity of 85%, specificity of 75%, and an AUC of 0.87 for predicting weaning failure [28]. Swathy Subhash also reported that a DTF greater than 20% was associated with extubation success, with a positive predictive value of 85% and an AUC of 0.771, indicating good diagnostic accuracy [12]. Similarly, Rui Li’s study reported a DTF sensitivity of 82.9%, specificity of 64.7%, and an AUC of 0.721 [29]. Our study reports these findings, with successfully weaned patients showing significantly higher DTF values. A DTF cutoff of ≤ 35.5% demonstrated a sensitivity of 70% and specificity of 75% (AUC = 0.789), suggesting its potential role in predicting weaning success.

Despite their diagnostic utility, ultrasound-based parameters are limited by operator dependency and may be influenced by anatomical variations, sedation levels, and underlying lung disease, all of which can affect measurement precision. Therefore, these parameters should be interpreted alongside clinical judgment and other physiological indicators to guide weaning decisions effectively [27, 30].

To improve the accuracy of weaning predictions, our study also explored combining diaphragmatic ultrasonographic parameters with the RSBI. The ratios of DE-RSBI and DTF-RSBI demonstrated superior predictive accuracy for weaning success compared to using RSBI alone. Patients who successfully underwent weaning exhibited significantly higher DE and DTF values, resulting in lower DE-RSBI and DTF-RSBI ratios. These ratios were strongly correlated with successful extubation.

ROC curve analysis revealed that these combined parameters offered superior sensitivity and specificity for predicting successful weaning. The DE-RSBI ratio showed the highest specificity, making it particularly effective for identifying patients at risk of weaning failure. Meanwhile, the DTF-RSBI ratio exhibited better sensitivity, enabling earlier detection of patients at risk for weaning failure. These findings suggest that incorporating diaphragmatic ultrasound measurements with RSBI could enhance the prediction of weaning outcomes and reduce failure rates. These findings align with prior research demonstrating the superior predictive value of ultrasound-derived indices compared to traditional metrics such as RSBI [32, 33].

In a study by Shamil et al., the ultrasound-derived diaphragmatic RSBI indices outperformed traditional RSBI, with a sensitivity of 90.2%, a specificity of 100%, and an AUC of 0.97. In contrast, RSBI showed a sensitivity of 40.5%, a specificity of 100%, and an AUC of 0.7, aligning with our findings [17]. Similarly, research by Hejia Ge et al. demonstrated that DE-RSBI had a sensitivity of 88.9%, a specificity of 81%, and an AUC of 0.853, while DTF-RSBI exhibited a sensitivity of 88.9%, a specificity of 85.7%, and an AUC of 0.7, further supporting our conclusions [34].

In our study, DE-RSBI with a cutoff value of > 1.56 and DTF-RSBI with a cutoff value of > 62.33 were statistically significant predictors of weaning success in univariate logistic regression analysis. However, these indices did not retain statistical significance in the multivariate model. This discrepancy may be due to the presence of confounding variables that were not adequately controlled for or may reflect the limited statistical power associated with the small number of weaning failure events (*n* = 10).

The lack of statistical significance in the multivariate analysis warrants cautious interpretation and highlights the multifactorial nature of weaning outcomes, which are often influenced by multiple overlapping factors. In contrast, Hejia Ge’s study, conducted in a pediatric population, demonstrated statistically significant results in multivariate analysis [34], although these may not be directly generalizable to adult ICU patients. Collectively, these findings emphasize the need for further research to refine measurement techniques and validate cutoff values across diverse patient populations.

### Limitation

This study has several limitations that should be acknowledged. First, the sample size was relatively small (*n* = 50), which limits the generalizability of the findings. Larger, multicenter studies are needed to validate the predictive value of DE-RSBI and DTF-RSBI and to establish standardized threshold values for broader clinical application.

Second, diaphragmatic ultrasound is inherently operator dependent. Although all measurements were performed by a single trained ICU physician to minimize variability, factors such as probe positioning, patient anatomy, and inter-operator experience can still influence measurement accuracy and reproducibility.

Third, this study was conducted in a single-center ICU, which may limit the external applicability of the findings to settings with differing patient demographics, weaning protocols, or ventilatory management strategies.

Fourth, potential confounding factors—such as fluid balance, nutritional status, and the use of sedatives or neuromuscular blocking agents—were not controlled for. These factors can significantly affect diaphragmatic performance and weaning outcomes.

Lastly, although DE-RSBI and DTF-RSBI demonstrated strong predictive accuracy in univariate analysis, their lack of statistical significance in multivariate analysis suggests the influence of unmeasured confounders and underscores the need for more robust model adjustment.

Future research should aim to address these limitations by including larger and more diverse patient populations, adjusting for additional confounding variables, and investigating the use of automated or AI-assisted ultrasound interpretation to enhance measurement standardization and clinical applicability.

## Conclusion

Diaphragmatic ultrasound-derived indices, such as DE-RSBI and DTF-RSBI, offer promising predictive value for weaning success in mechanically ventilated patients. Compared to the traditional RSBI, these composite measures demonstrate enhanced sensitivity and specificity. However, given their dependence on operator technique and the multifactorial nature of weaning failure, larger multicenter trials are necessary to validate their clinical utility and establish standardized cutoff values.

## Data Availability

data is available on request.
